# Electric Surgery

**Published:** 1885-01

**Authors:** 


### Electrical Surgery.

A few days ago a boy named Ulmer, in Portland, Me., got a piece of steel into one of his hands. A surgeon examined the wound and decided that he would have to cut the hand open for two inches to extract the piece of steel. He first took the boy to the Western Light station, and tried the value of electricity as a surgical aid. The piece of steel had gone down through the hand. A steel instrument was inserted into the wound until it reached the piece and the instrument was then magnetized, and drawn from the wound, drawing the piece with it, leaving only the small hole where it entered to heal, and thereby saving the usefulness of the hand.

